# Effect of foliar application of selenium on morphological and physiological indices of savory (*Satureja hortensis*) under cadmium stress

**DOI:** 10.1002/fsn3.1943

**Published:** 2020-10-18

**Authors:** Iraj Azizi, Behrooz Esmaielpour, Hamideh Fatemi

**Affiliations:** ^1^ Department of Horticulture University of Mohaghegh Ardabili Ardabil Iran

**Keywords:** cadmium, heavy metals, savory, selenium

## Abstract

Cadmium is a heavy metal that pollutes the environment and affects plants physiologically and morphologically. Selenium is considered as a beneficial element, with effective roles in increasing plant tolerance to environmental stresses. A greenhouse factorial pot experiment was conducted to study the impact of selenium on traits of Savory plants under Cd stress. Experimental factors included soil contamination with cadmium (0, 75, 100, and 150 μM) and foliar spraying of selenium (0, 10, 20, and 40 μM of Sodium Selenate). Biomass, photosynthetic pigments including chlorophyll a, chlorophyll b, total chlorophyll, proline, total soluble solids, cell membrane leakage, relative water content of leaves antioxidant enzymes, and Cd and Zn concentration in shoot and root were recorded. Results revealed that Cd stress decreased vegetative growth criteria, photosynthetic pigments include chlorophyll a, chlorophyll b, total chlorophyll, and carotenoid almost, 55%, 57%, 57%, and 68%, respectively, while poline, cell membrane leakage, peroxidase (POD), and catalase (CAT) antioxidant enzymes were increased with increasing Cd concentrations. Foliar spray of selenium reduced the toxic effects of Cd stress on savory plants via enhancing of proline content and stimulation of CAT and POD enzymes and limitation of cell membrane leakage. Also, selenium foliar spray improved chlorophyll content under Cd stress condition and decreased cadmium accumulation 29% in root, respectively. In general, these results suggest that foliar application of selenium could mitigate Cd toxicity and improve growth and antioxidant capacity of savory under different level of cadmium heavy metal stress.

## Graphical abstract

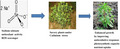

## 1 INTRODUCTION

Cadmium is one of the most toxic heavy metals among major environmental pollutants. Cd is released into water and soil by humans through urban, industrial, and agricultural activities. Most importantly, contamination by Cd in agriculture happens through long‐term use of phosphate fertilizers, contaminated water, and waste water application in irrigation (He et al., 2009; Uraguchi & Fujiwara, 2012). This unnecessary element which is considered as a highly mobile contaminant is allowed to enter into vegetables easily and can enter into several vital processes of the plant, thereby leading to poor growth, low economic performance of plants, and threats to human health (Di Toppi & Gabbrielli, 1999; Ekmekçi et al., 2008; Shamsi et al., 2008).

Cd‐contaminated agricultural land is a major problem because the metal is easily absorbed by the root of the plants and can be translocate to aerial parts. Cd can impair the process of water absorption and cause an imbalance in micronutrient content, photosynthesis, and nitrogen metabolism. Ultimately, it inhibits plant growth and reduces the biomass (Anjum et al., 2008; Ghaghelestany et al., 2020; Gill et al., 2012; Jahanbakhshi & Kheiralipour, 2019). It can lead to plant death in severe cases of contamination (Di Toppi & Gabbrielli, 1999). Cd can inhibit root and branch growth, and reduce chlorophyll biosynthesis (Siedlecka & Krupa, 1999). It usually disrupts photosynthesis, respiration, and relative water content (Gouia et al., 2003). Several studies confirmed that the effects of Cd on decrease in activity of responsible enzymes in the absorption of nitrate and sulfate in plants (Ghnaya et al., 2005; Gouia et al., 2003). Cd can prevent the activity of enzymes which play important roles in the Calvin cycle in Sandalio et al., (2001), the synthesis of carbohydrates, and phosphorus metabolism (Di Toppi & Gabbrielli, 1999).

Cd stimulates the generation of reactive oxygen species (ROS) and leads to oxidative stress in most plants (Gill & Tuteja, 2010) which disrupt photosynthetic pigments and biomolecules such as lipids, proteins, and nucleic acids, along with a significant reduction in growth, production, and even the death of a plant (Foyer & Noctor, 2005). Therefore, plants have evolved antioxidant defense to ameliorate oxidative damage of Cd stress via enzymatic antioxidants (SOD, CAT, APX and GR), nonenzymatic antioxidants (glutathione [GSH] and ascorbate [AsA]; Gill et al., 2012; Mittler et al., 2004).

Selenium is an essential micronutrient with antioxidant, anti‐cancer, and antiviral properties for the health of humans and animals, although the need for Se in plants is not proven as yet (Pilon‐Smits, 2015). Se at low concentrations plays an important role in antioxidant reactions such as increased glutathione peroxidase activity and hormonal balance in plant cells (Cartes et al., 2010; Filek et al., 2008). The use of low levels of Se (5 and 10 μM) increased growth and photosynthetic capacity of treated cucumber seedlings under NaCl salinity (Hawrylak‐Nowak, 2009).

Selenium plays protective and antioxidant role in decreasing oxidative stress caused by temperature, drought, salinity, mechanical stress, UV radiation, pathogens, and heavy metals. Also, selenium ameliorates stress by increasing the antioxidant capacity of the plant through increasing the activity of enzymatic antioxidants and nonenzymatic antioxidants (Ahangarnezhad et al., 2019; Azarmdel et al., 2020; Cartes et al., 2010; Haghighi et al., 2016; Jahanbakhshi et al., 2018, 2019, 2020; Lin et al., 2012; Momeny et al., 2020; Pandey & Gupta, 2015; Qing et al., 2015; Yao et al., 2009).

Summer savory (*Satureja hortensis* L.) is an annual and herbaceous plant from Lamiaceae family (Mumivand et al., 2013). The leaves and shoots of summer savory contain essential oil (Omidbaigi, 2009), and its essential oil is being used widely in medicinal, food, and health industrials (Leake et al., 2003). Summer savory is also used in the traditional medicine to treat muscle pains, indigestion, diarrhoea, and infection diseases (Gursoy et al., 2009). The major essential oil compounds of summer savory are carvacrol, thymol, γ‐terpinene, and borneol (Kamkar et al., 2013).

Since summer savory is an important medicinal crop, it would be valuable to investigate the responses of this plant to Cd stress. Foliar spraying of Se may alleviate some of the detrimental effects of Cd on *Satureja hortensis* plants. Therefore, this research was conducted to investigate the impacts of foliar spray of Se on some physiological and biochemical characteristics of summer savory under Cd stress.

## 2 MATERIALS AND METHODS

### 2.1 Experimental design and soil preparation

A pot experiment was arranged as factorial on the basis of completely randomized design with three replicates in the greenhouse of the Agricultural and Natural Resources Faculty of University of Mohaghegh Ardabili to assess the response of Se foliar spraying on growth and physiology of summer savory (*Satureja hortensis* L.) under Cd stress condition. Cadmium chloride (CdCl_2_) and sodium selenate (Na_2_SeO_4_) were purchased from Merck. The CdCl_2_ solution was spiked as Cd stress in four concentrations (0, 75, 100, and 150 µM) to soil, and its moisture content was adjusted to field capacity by adding deionized water. The soils were incubated in stable (darkness, 40°C, in dry and wet) conditions for four months with frequent stirring to allow complete equilibration.

### 2.2 Seedling cultivation

**Figure 1:**
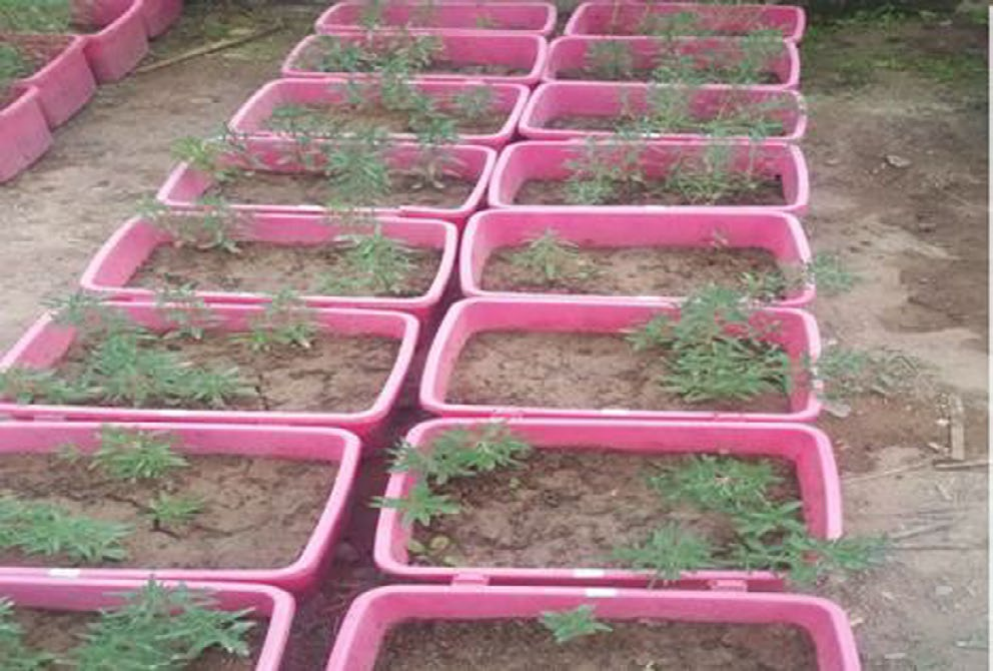
Savory seedlings after foliar application

The seeds of Summer savory native ecotype of Shahr ray were obtained from the Medicinal Plant Research Station of Shahid Beheshti University of Tehran province, Iran. These seeds were surface sterilized with sodium hypochlorite for 5 min and washed five times with distilled water. Savory seeds (*Satureja hortensis*) were planted at a depth of 0.5–1 cm in pots containing 10 kg Cd‐polluted soil. Pots then kept in greenhouse at growth conditions consisting the temperature of 22°C, relative humidity of 40%–50%, and average light intensity of 50% (Figure 1). Sodium selenate at four concentrations (0, 10, 20, and 40 µM) was applied for foliar spray in three growth stage. The first treatment was applied after emergence of two true leaves, and the other two foliar spraying were applied at 2‐week intervals.

### 2.3 Traits measurement

At the end of growth stage (4 months, at flowering stage), the plants were removed and separated into different parts (root, shoot, and leaf), then their morphological traits such as number of leaves, number of lateral branches, plant height by using meter rod, plant biomass (weighing balance), and root weight (weighing balance) after oven drying for 72 hr at 70°C.

### 2.4 Photosynthetic pigments

Photosynthetic pigments and carotenoid were extracted from fresh leaves according to the method of Harmut (1987). About 0.1 g of leaf homogenated with acetone (80%). The absorbance of the pigments was measured by UV visible spectrophotometer (Jenway, Italy) 470, 645, and 663 nm. Photosynthetic pigments and carotenoid content were estimated using the equations proposed by Harmut (1987).

### 2.5 Membrane stability index

For determination of membrane stability index, leaf disks were prepared from fully expanded leaves and were washed three times with deionized water. The samples were placed in container containing 10 ml deionized water for 24 hr and shaked at 25°C. Then, the EC value of each sample was measured (Lt). The samples and solution were autoclaved then EC was measured (L0). The electrolyte leakage from cell membrane was estimated by Equation (1) proposed by Redman et al. (1986).

(1) $$\text{Soluble}\;\text{materials}\;\text{leakage}\;\%=\frac{L_{t}}{L_{0}}\times 100$$

### 2.6 Relative water content

Relative water content of plants was determined at the end of experiment according to Ritchie et al. (1990) method. For instance, 0.5 g from the youngest leaf of each plant (FW) was sampled and floated in distilled water for 24 hr then leaf saturation weight was measured (TW). Finally, the leaves were *n* placed in an oven at 70°C for 24 hr, and their dry weight was measured according to following equation:

(2) $$\text{RWC}\%=\frac{\text{FW}‐\text{DW}}{\text{TW}‐\text{DW}}$$

### 2.7 Proline accumulation

Proline content of leaves was determined by method of Bates et al. (1973). Samples from the youngest leaves (0.5 g) in each treatment was homogenized in 2 ml of 3% sulfosalicylic acid and then was centrifuged at 2000 *g* for 5 min. The filtered homogenate was mixed with equal volume of ninhydrin and glacial acetic acid in a test tube within a water bath at 100°C for an hour. The reaction was terminated in an ice bath, and the solution was extracted by toluene. The absorbance was recorded at 520 nm. The proline content was calculated according to standard curves of L‐proline and reported as μg per g leaf fresh weight.

### 2.8 Measurement of carbohydrate content

In order to measure the amount of carbohydrates, at first the extract of the leaves was prepared. For this purpose, 0.1 g of leaf specimen, along with 1 ml ethanol (95%), was pulverized inside a porcelain mortar. Then, 1 ml ethanol (70%) was added to it. The final solution was centrifuged at 2058 *g* for 15 min. After removing the alcoholic extract containing soluble sugars, 0.1 mg of this extract was added to 3 mg antiroll‐sulfuric acid (72%). The mixture was placed in a water bath for 15 min at 100°C. After cooling the mixture, the amounts of soluble sugars were measured at 625 nm (Irigoyen et al., 1992).

### 2.9 CAT enzyme activity

Protein extraction was carried out based on Sudhakar et al. (2001). Catalase activities (CAT) were estimated using the procedure described by Kar and Mishra (1976). For the catalase enzyme, 60 μl of protein extract was added to 2.5 ml 50 mM buffer with pH = 7 and 0.3 ml 5 mg oxygenated water in an ice bath. The curve of absorption and variation was read at 240 nm. Enzyme activity (μg/ml) was measured of fresh protein.

### 2.10 POD enzyme activity

The activity of peroxidase was determined by guaiacol and H_2_O_2_ substrates as described by Chance and Maehly (1955). With the extinction coefficient of tetraguaiacol product (26.6 mM^−1^ cm^−1^), the activity of POX was expressed as mmol produced tetraguaiacol per minute per mg soluble protein (U/mg).

### 2.11 Measurement of cadmium and zinc elements

Leaf samples from each treatment rinsed with deionized water and then dried at 65°C for 48 hr. The samples were powdered uniformly. Then, (0.1 g) from plant samples were digested with an acid mixture of HNO_3_/HClO_4_ at 100°C and placed in a furnace at 550°C for 5 hr. After cooling, the specimens were removed from the furnace and 10 ml of 2 N normal chloride acid was added to it. The samples were dissolved in acid with a gentle heat. The solution was filtered through a filter paper (Whatman filter paper No. 42) and was poured into a 50cc laboratory flask. Distilled water was added to a filter paper to wash the remaining material in the funnel in order to transfer the extract back into the laboratory flask. Finally, the extract was diluted with water and reached a volume of 50cc. It was stored for chemical analysis in the refrigerator (Jones, 2001). The amounts of Cd and Zn in the extracts of the aerial parts and roots were read separately by atomic absorption spectrometers.

### 2.12 Statistical analysis

All data were subjected to two‐way analysis of variance (ANOVA) in SAS 9.1. The mean values were separated by LSD (Least Significant Difference) test at p_0.05 (Jahanbakhshi et al., 2020). The *p* values of less than .05 considered statistically significant.

## 3 RESULTS

### 3.1 Morphological traits

Cd stress, Se foliar spray, and their interactions significantly influenced biomass (Table 1). Increasing Cd stress resulted in continuous reductions of vegetative traits such as stem height, fresh and dry weight of root, dry weight of plant, and number of lateral leaves and branches. Se foliar spraying significantly improved the vegetative traits of summer savory plants under different concentrations of Cd stress. According to the results of the, the highest value for all of morphological traits was obtained by foliar spraying of 40 μM Se in plants grown in unpolluted soils and the least amount of these traits was related to 150 μM of Cd and Se control treatment. It seems that Se has increased the amount of carbohydrates, dry and wet weight, and other morphological characteristics of the plant by improving the process of photosynthesis and reducing the damage to chlorophyll under Cd stress.

**Table 1:** Comparison of the interactions between selenium soluble and cadmium stress on morphological indices of savory

Treatment (µM)	Stem height (cm)	Root fresh weight (g)	Number of lateral branches	Number of leaves	Dry plant weight (g)	Root dry weight (g)
Cd 0						
Se 0	45.83^bc^	2.21^abc^	22.50^bc^	146.67^abcd^	4.90^abcd^	0.65^ab^
Se 10	47.83^abc^	2.52^ab^	24.16^ab^	163.33^ab^	5.46^ab^	0.82^ab^
Se 20	49.16^ab^	2.53^ab^	25.66^ab^	172.33^a^	6.02^a^	0.90^a^
Se 40	50.66^a^	2.76^a^	27.33^a^	173.33^a^	6.02^a^	0.90^a^
Cd 75						
Se 0	39.00^efg^	1.70^bcd^	21.66^bcd^	131.67^abcdef^	4.59^abcde^	0.65^ab^
Se 10	39.83^ef^	1.80^bcd^	21.83^bcd^	145.00^abcde^	4.25^bcdef^	0.66^ab^
Se 20	41.16^de^	2.09^abc^	22.33^bcd^	146.67^abcd^	5.37^abc^	0.69^ab^
Se 40	44.50^dc^	2.21^abc^	22.33^bcd^	161.33^abc^	5.40^abc^	0.76^ab^
Cd 100						
Se 0	37.00^efg^	1.42^dc^	20.50^cde^	105.00^def^	2.85^f^	0.56^bc^
Se 10	37.66^efg^	1.45^dc^	21.00^cd^	116.67^bdef^	3.79^cdef^	0.56^bc^
Se 20	38.33^efg^	1.48^dc^	22.16^bcd^	116.67^bdef^	4.13^bcdef^	0.57^bc^
Se 40	39.16^ef^	1.63^bcd^	22.00^bcd^	126.67^abcdef^	4.28^bcdef^	0.61^abc^
Cd 150						
Se 0	34.66^e^	1.08^d^	18.00^e^	95.00^f^	2.85^f^	0.33^c^
Se 10	35.66^fg^	1.34^dc^	19.00^e^	98.33^ef^	3.14^ef^	0.33^c^
Se 20	35.83^fg^	1.42^dc^	19.50^de^	105.00d^ef^	3.25^ef^	0.54b^c^
Se 40	36.16^fg^	1.42^dc^	20.50^cde^	115.00cd^ef^	3.58^def^	0.55b^c^

The alphabets in each column do not show statistically significant difference in the 5% probability level based on the LSD test.

Nonsimilar letters indicate a significant difference at 5% probability level.

### 3.2 Proline

The results show that Proline accumulation significantly increased with increasing the Cd stress in savory plants (Table 2). Likewise, increasing the concentration of Se after foliar application increases the amount of proline, so that the highest proline content 1.82 µg(FW)^−1^ is achieved by 150 μM Cd contamination and 40 μM Se treatment.

**Table 2:** Comparison of the interactions between selenium soluble and cadmium stress on Physiological indices of savory

Treatment (µM)	Proline [µg(FW)^−1^]	Carbohydrate [µg(FW)^−1^]	Chlorophyll A [µg(FW)^−1^]	Chlorophyll b [µg(FW)^−1^]	Total chlorophyll [µg(FW)^−1^]	Carotenoid [µg(FW)^−1^]	Membrane cell leakage (%)
Cd 0							
Se 0	0.41^h^	0.29^g^	6.45^d^	2.42^c^	9.18^c^	1.69^de^	13.30^fgh^
Se 10	0.46^gh^	0.33^fg^	7.19^bc^	2.46^c^	9.81^b^	1.92^bc^	12.61^ghi^
Se 20	0.52f^gh^	0.37^fg^	7.33^b^	2.81^ab^	10.15^b^	2.19^ab^	11.74^ij^
Se 40	0.6^fg^	0.44f^g^	8.18^a^	3.04^a^	11.23^a^	2.38^a^	10.97^j^
Cd 75							
Se 0	0.43^h^	0.35^fg^	5.6^ef^	1.76^efg^	7.33^f^	1.29^fg^	15.33^de^
Se 10	0.55f^gh^	0.42^fg^	6.16^de^	1.83^def^	8.14^e^	1.47^ef^	14.33^ef^
Se 20	0.66^f^	0.5^ef^	6.47^d^	1.95^de^	8.48^de^	1.56^e^	13.48^fg^
Se 40	0.87^e^	0.65^dce^	6.71^cd^	2.01^d^	8.87^cd^	1.83^cd^	11.92^hij^
Cd 100							
Se 0	0.82^e^	0.47^ef^	4.69^gh^	1.47^hij^	5.83^hi^	0.84^ij^	18.33^b^
Se 10	1.04^d^	0.62^ef^	4.89^gh^	1.83^def^	6.83^fg^	0.94^hij^	16.72^cd^
Se 20	1.28^c^	0.8^cd^	5.05^fg^	1.59^ghi^	6.84^fg^	1.01^h^	14.43^ef^
Se 40	1.31^c^	1.03^b^	5.49^f^	1.67^fgh^	7.27^f^	1.15^gh^	12.42^ghij^
Cd 150							
Se 0	1.02^d^	0.64^de^	2.87^j^	1.04^k^	3.91^j^	0.54^k^	20.65^a^
Se 10	1.23^c^	0.83^de^	3.89^i^	1.33^j^	5.29^i^	0.71^jk^	18.19^b^
Se 20	1.52^b^	1.09^b^	4^i^	1.41^ij^	5.83^hi^	0.94^hji^	16.19^bc^
Se 40	1.82^a^	1.39^a^	4.35^hi^	1.47^hij^	6.29^gh^	1.08^ghi^	15.08^e^

The alphabets in each column do not show statistically significant difference in the 5% probability level based on the LSD test.

Nonsimilar letters indicate a significant difference at 5% probability level.

### 3.3 Carbohydrate

The results state that (Table 2), the highest amount of carbohydrates 1.39 µg (FW)^−1^ is seen in the treatment of 40 μM Se and the contamination level is 150 μM Cd and the lowest amount is in the control level of Se and Cd.

### 3.4 Chlorophyll

According to the results of comparison of mean (Table 2) of the interaction effect of Cd stress and foliar application of Se on this index, the chlorophyll content (a, b, and carotenoids) also decreased by increasing Cd concentration, but increased Se concentration increases chlorophyll content (a, b, and carotenoids). So that, the highest levels of chlorophyll a, b, and carotenoids are related to the level of Cd without contamination and 40 μM Se treatment.

### 3.5 Membrane cell leakage

Evidence suggests (Table 2) that increasing the concentration of Cd leads to disruption of membrane protein pumps and with increasing concentration of Cd ion leakage from the cell increases but the application of Se reduces cell leakage. So that, the highest membrane leakage rate 20.65 (%) is related to 150 μM Cd contamination and Se control treatment, and the lowest membrane leakage rate 10.97(%) is related to the level of non‐contamination Cd and 40 μM treatment of Se.

### 3.6 Antioxidant enzymes

As Cd stress increases compared to the control level, oxidative stress also increases, which leads to the production of reaction oxygen species (ROS), and the plant's antioxidant system is activated to counteract this stress, and Se reduces this stress by improving this activity, as seen the highest amount of POD and CAT enzymes is related to 150 μM Cd contamination and 40 μM Se treatment and the lowest amount of POD enzyme is related to noncontamination level of Cd and control treatment of Se (Table 3).

**Table 3:** Comparison of the interactions between selenium soluble and cadmium stress on antioxidant enzymes of savory

Treatment (µM)	POD enzyme [µg protein (min)^−1^]	CAT enzyme [µg protein (min)^−1^]
Cd 0		
Se 0	0.00106^i^	0.000150^g^
Se 10	0.00126^hi^	0.000163^g^
Se 20	0.00143^gh^	0.000183^fg^
Se 40	0.00160^efg^	0.000210^ef^
Cd 75		
Se 0	0.00126^hi^	0.000180^fg^
Se 10	0.00146^fgh^	0.000203^f^
Se 20	0.00170^ef^	0.000243^e^
Se 40	0.00196^cd^	0.000286^d^
Cd 100		
Se 0	0.00140^gh^	0.000243^e^
Se 10	0.00173^de^	0.000296^d^
Se 20	0.00206^cd^	0.000343^c^
Se 40	0.00250^b^	0.000393^b^
Cd 150		
Se 0	0.00173^de^	0.000303^d^
Se 10	0.00203^c^	0.000350^c^
Se 20	0.00243^b^	0.000416^b^
Se 40	0.00283^a^	0.000473^a^

The alphabets in each column do not show statistically significant difference in the 5% probability level based on the LSD test.

Nonsimilar letters indicate a significant difference at 5% probability level.

### 3.7 Relative water content of leaves

**Figure 2:**
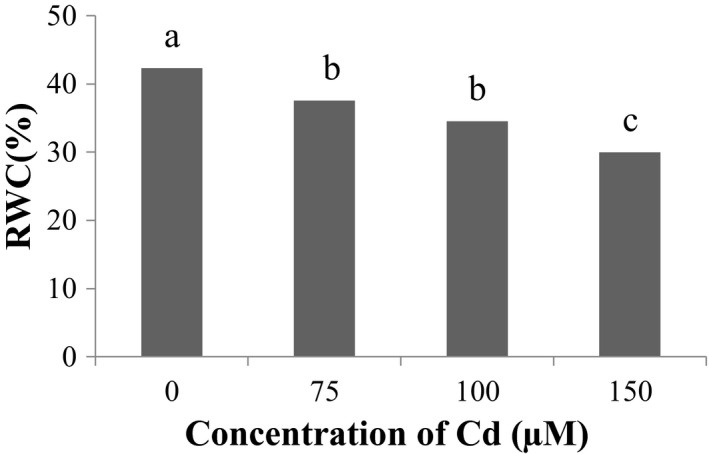
Effect of cd stress on leaf relative water content

**Figure 3:**
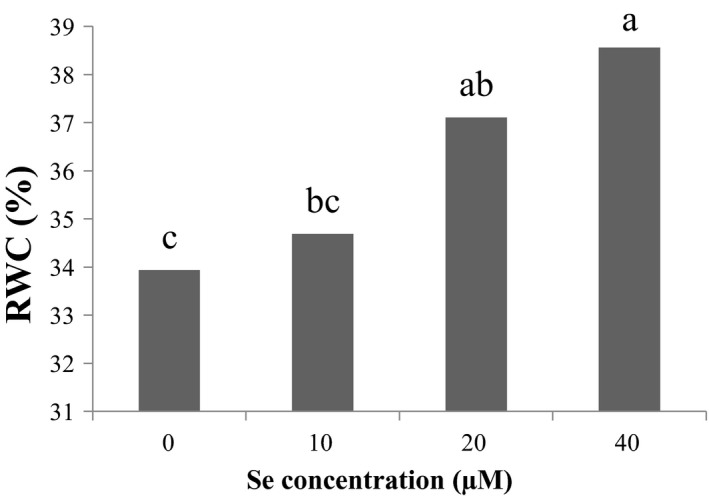
Effect of Se on leaf relative water content

According to the results of (Figure 2) of the effect of Cd stress on relative water content of leaves, the most percentage of relative water content was related to the control level of Cd and the lowest relative water content was related to 150 μM Cd. The relative water content of leaves decreases by increasing the concentration of Cd. But Se maintains the relative water content of the leaf by affecting the leaf cell stomata, so that the highest relative water content is related to 40 μM Se (Figure 3).

### 3.8 Aerial parts Cd

**Figure 4:**
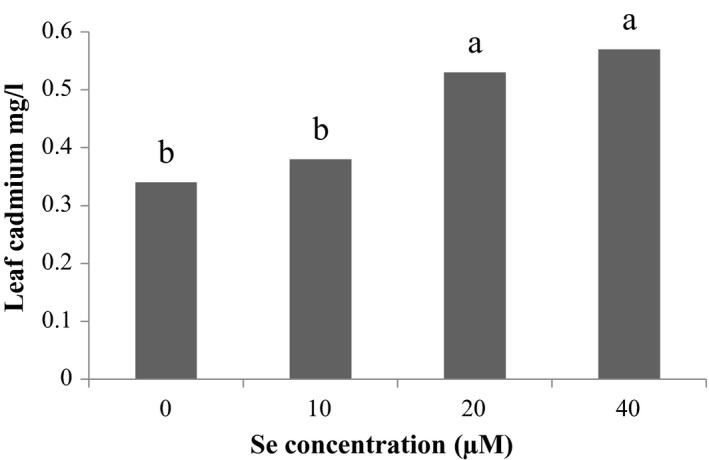
Effect of Se on Aerial Cd

**Figure 5:**
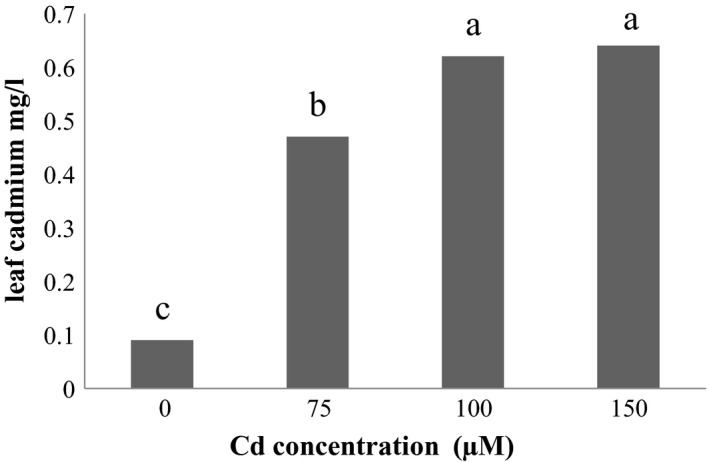
Effect of Cd stress on Aerial Cd

Studies on the effect of Se foliar application on Cd accumulation in aerial parts of the plant showed that the most element of aerial part Cd was observed in 40 μM Se treatment and the least amount was related to control Se treatment (Figure 4). The results also showed the effect of Cd stress on the Cd aerial part, the highest accumulation of Cd in the aerial part is related to Cd 150 μM and the lowest amount of Cd aerial part is related to the Cd control level (Figure 5).

### 3.9 The accumulation of root Cd

In this study (Table 4), the highest root Cd accumulation 1.92 mg/L was in 150 μM Cd and Se control and the lowest root Cd concentration 1.07 mg/L was related to the control Cd and 40 μM Se treatment.

**Table 4:** Comparison of the interactions between selenium soluble and cadmium stress on morphological indices of savory

Treatment (µM)	Root cadmium (mg/L)	Leaf zinc (mg/L)
Cd 0		
Se 0	1.28^efg^	1.30^ab^
Se 10	1.13^gh^	1.30^ab^
Se 20	1.07^h^	1.33^ab^
Se 40	1.07^h^	1.42^a^
Cd 75		
Se 0	1.29^efg^	1.33^ab^
Se 10	1.21^efgh^	1.31^ab^
Se 20	1.51^bcd^	1.21^abc^
Se 40	1.33^def^	1.09^bcd^
Cd 100		
Se 0	1.54^bc^	0.86^defg^
Se 10	1.51^bcd^	0.96^cdef^
Se 20	1.36^cde^	1.09^bcd^
Se 40	1.33^def^	1.02^bcde^
Cd 150		
Se 0	1.92^a^	0.14^h^
Se 10	1.68^b^	0.51^g^
Se 20	1.39^cde^	0.61^fg^
Se 40	1.36^cde^	0.83^efg^

The alphabets in each column do not show statistically significant difference in the 5% probability level based on the LSD test.

Nonsimilar letters indicate a significant difference at 5% probability level.

### 3.10 Zinc element accumulation on the aerial parts

Due to the chemical similarity of Cd to zinc, Cd replaces this element. Therefore, the lowest accumulation of zinc 14 mg/L is observed in the 150 μM Cd treatment and the highest accumulation 1.42 mg/L is observed in the control surface of Cd and 40 μM Se treatment (Table 4).

### 3.11 Zinc element accumulation on the root

**Figure 6:**
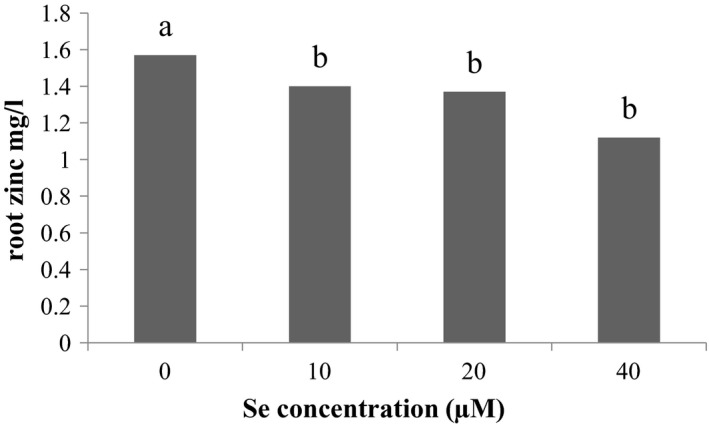
Effect of Se on root zinc

**Figure 7:**
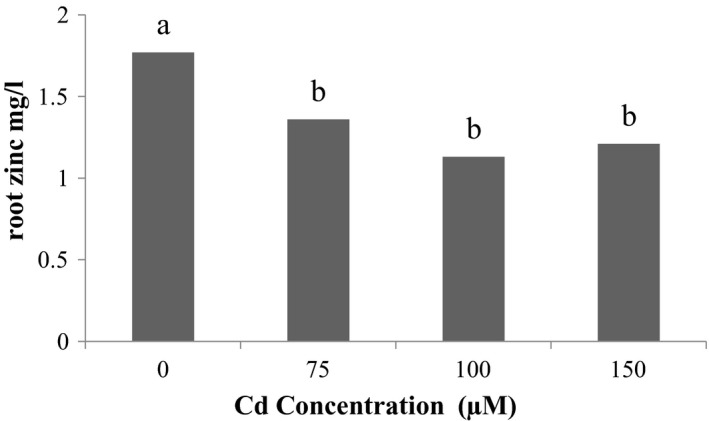
Effect of Cd on root zinc

Examination showed the accumulation of zinc on the roots (Figure 6), the most amount of zinc accumulation on the root was related to Se control treatment and the least amount of element on the root is related to 40 µM Se treatment. According to the results (Figure 7) of the effect of Cd stress on root zinc, the most amount of accumulation on the root is related to the control level of Cd and the least root level is related to 150 μM Cd.

## 4 DISCUSSION

Cadmium is considered as a heavy element in the Earth's crust. It is not only unnecessary for the physiological processes of plants but is also toxic to plants and animals even in very low quantities. Reduction in growth and chlorosis is considered as primary effects of Cd on plants. They lead to fewer stems, smaller leaf area, and lower values of fresh and dry weight of the root and other vegetable parts. Some of the most important causes of growth impairment by heavy metals include disorders in the cellular water balance and cell wall elasticity, disorders in the plant's water balance due to reduced cell size, and fewer xylems, along with a reduced absorption of essential nutrients such as Fe^+2^, K^+^, Mg^+2^, and Ca^+2^. Heavy metals can reduce the growth of plants significantly by severely disrupting photosynthesis, by deterring photosynthetic production and cell division. These ultimately result in shorter internode length and shorter plant height (Chaffei et al., 2003). The interaction of selenite with the plasma membrane, along with changes in membrane permeability regarding ions (potassium, sodium, and calcium), may affect plant growth, respiration, water uptake, and phloem activity (Dziubinska et al., 2010). Also, Se is a component of several amino acids. It increases the production of ethylene and promotes changes in the composition of membrane lipids, while increasing membrane permeability, potassium leakage, and water content in the intercellular space (Xue et al., 2001).

Proline can act as an antioxidant to reduce the threat of free radicals. It maintains the integrity of the membrane by inhibiting lipid peroxidation (Mehta & Gaur, 1999). In general, there is a special relationship between proline accumulation and water deficiency caused by heavy metals, which ultimately inhibits plant growth (Metwally et al., 2003). There could be a significant relationship between the accumulation of proline under Cd treatment with mechanisms of resistance to osmotic changes. This relationship could also mean a decrease in the activity of the electron transfer system in plants or parts of the plant. Also, a decrease in metabolic activity leads to the accumulation of NADH. Since 2NADH molecules are required for synthesis of a proline molecule of glutamic acid, proline synthesis can serve as a mechanism to reduce acidity and prevent the accumulation of NADH (Saradhi, 1991).

According to previous results (John et al., 2008), the amount of soluble and insoluble carbohydrates in plants can increase or decrease depending on the concentration of heavy metals where the plants grow. Soluble sugars increase by changing the activity of protein channels of water transfer and by closing the leaf stomata, thereby limiting the loss of water from the plant (Zhang & Tyerman, 1999). By decreasing the transfer of water into leaves and after the accumulation of Cd in the cells, the amount of soluble sugars in the plant increases. In addition, the increase in soluble sugars helps the plant keep its carbohydrate content in order to maintain basal metabolism under stress conditions. These results are consistent with a previous report on rice (Verma & Dubey, 2001).

According to the results of this study, the amounts of chlorophyll and carotenoids in savory seedlings decreased when increasing the Cd concentration in the growth medium. Heavy metals disrupt compound formations by inhibiting the biosynthesis of LHCII compound proteins at the transcriptional level (Katznelson et al., 1962). Carotenoids play an important role in protection against oxidative stress. These pigments can assist in detoxification and can reduce the toxic effects of free radicals (Di Toppi & Gabbrielli, 1999).

A low amount of chlorophyll content in the leaf can occur when the synthesis of photosynthetic pigments is inhibited by Cd. This happens because of the lowered absorption of essential nutrients such as iron, manganese, and magnesium (Prasad & Strzałka, 1999). Increasing the amount of Se to appropriate levels can partially reduce chloroplast degradation and increase chlorophyll content (Cai et al., 2011).

Liu et al. (2004) reported that the reaction of Se with ROS can reduce the negative effects of Cd on chlorophyll content in rice leaves. This is caused because free oxygen radicals can break down photosynthetic pigments and structural proteins of the photosynthetic system under Cd stress conditions. This has been proven especially on D1 protein (i.e., one of the first targets for oxidative degradation in the photosystem 2 reaction center; Kim et al., 2006). The restoration of photosynthesis in plants under stress after application of Se may be associated with reduced levels of ROS (Paciolla et al., 2011).

Heavy metals can damage the membrane by disabling membrane enzymes and inhibiting membrane ATPase activity, thereby affecting membrane integrity (Ruley et al., 2006). Heavy metals cause changes in the cell membrane lipid composition. Thus, damages to the cell membrane can lead to the release of ions out of the cell (Singh et al., 2010). In this study, increasing the cadmium concentration enhanced the amount of ion leakage. The highest ion leakage rate occurred in response to the 150 μM Cd treatment. Meanwhile, Se reduced the ion leakage and increased cell membrane stability by increasing the potassium uptake in plant cells (SaffarYazdy et al., 2012). The lowest amount of ion leakage resulted from using 40 μM Se with no amount of Cd treatment.

It has been proven that peroxidase acts as the main enzyme against stresses caused by heavy metals. This enzyme is known as a stress marker in conditions of heavy metal stress (Choudhary et al., 2007). The formation of ROS can be controlled by the presence of antioxidant enzymes, including peroxidases in plant cells. These stimulate the antioxidant activity in aerial parts of plants and roots where soils contain heavy metals. This is in accordance with previous reports on *Solanum lycopersicum* (Quiroga et al., 2000) and *Raphanus sativus* (Chen et al., 2002).

In this study, observations show that the highest accumulation of cadmium in the roots occurred by 150 μM Cd. In this regard, ions are transferred through the cell membrane by unique proteins called transporters. Of the total ions around the root, only a small part is absorbed by the plant. Most of these ions are physically absorbed by the cell wall where the –COO compartment has a negative charge and is responsible for surface absorption in the cell wall. Ions that attach to this compartment cannot enter the cell in the aerial parts of plants. The accumulation of these elements increases the concentration of Cd in cellular vacuoles and prevents their transfer to aerial parts. Therefore, the amount of this element in roots is greater than its amount in aerial parts (Ramos et al., 2002).

Cd is chemically similar to zinc. It imitates the metabolic functions of zinc in the plant (Mengel & Kirkby, 2001) and can be absorbed into the plant instead of zinc (Grant et al., 1998). This similarity in the Cd and zinc properties indicates the importance of their interaction in absorption. It highlights the transfer from roots to the aerial part and the accumulation of Cd in edible tissues, as this element ultimately enters the food chain (An et al., 2005). In this study, it seems that Cd replaced zinc in the aerial parts and that the lowest amount of zinc was observed as a result of the 150 μM Cd treatment.

## 5 CONCLUSION

Cadmium stress reduced photosynthesis and cell division. It affected the quality of morphological indicators in savory seedlings, whereas spraying selenium on the seedlings reduced the rate of chloroplast destruction. In fact, selenium increased the amount of chlorophyll to some extent and alleviated the toxic effects of stress caused by cadmium. Nutritional supplementation with selenium enhanced several biochemical features such as proline, carbohydrate, and chlorophyll in savory seedlings under cadmium stress conditions. Selenium reduced cell leakage and increased the activity of catalase and peroxidase enzymes. The treatment worked best when selenium was applied at a concentration of 40 μM. Spraying the selenium solution on plants reduced the accumulation of cadmium in the roots and increased the uptake of zinc, followed by the translocation of zinc to the aerial parts of the plant. Therefore, selenium can be an effective agent against the toxic effects of cadmium.

## CONFLICT OF INTEREST

The authors have declared no conflict of interest.

## ETHICAL APPROVAL

This study does not involve any human or animal testing.

## INFORMED CONSENT

Written informed consent was obtained from all study participants.

## Data Availability

Due to the nature of this research, participants of this study did not agree for their data to be shared publicly, so supporting data are not available.
